# Measuring Ethnic Preferences in Bosnia and Herzegovina with Mobile Advertising

**DOI:** 10.1371/journal.pone.0167779

**Published:** 2016-12-22

**Authors:** Annerose Nisser, Nils B. Weidmann

**Affiliations:** 1 Department of Politics and Public Administration, University of Konstanz, Konstanz, Germany; 2 Graduate School of Decision Sciences, University of Konstanz, Konstanz, Germany; University of Rijeka, CROATIA

## Abstract

We present a field experiment that uses geo-referenced smartphone advertisements to measure ethnic preferences at a highly disaggregated level. Different types of banners advertising a vote matching tool are randomly displayed to mobile Internet users in Bosnia and Herzegovina, while recording their spatial coordinates. Differences in the response (click) rate to different ethnic cues on these banners are used to measure temporal and spatial variation in ethnic preferences among the population of Bosnia and Herzegovina. Our study lays out the theoretical and practical underpinnings of this technology and discusses its potential for future applications, but also highlights limitations of this approach.

## Introduction

Existing research has shown that ethnic diversity may negatively affect economic performance and political stability [1–4]. It has been argued that a co-ethnic bias is one of the underlying mechanisms explaining this relationship. A co-ethnic bias is a preferential treatment of members from one’s own ethnic group vis-à-vis those of other groups in economic, social and political interactions [5]. A number of prominent studies have examined the existence of, and contributing factors to, this co-ethnic bias [1, 6]. However, those studies rely mostly on resource-intensive lab-in-the field settings. Although this approach has clear advantages, experiments of this type have high costs for set-up and execution, and are necessarily limited in scope. Is there variation in co-ethnic bias across a country? Does its strength, and possibly also its direction, vary? Existing experimental approaches cannot tell. In this study, we aim to tackle these limitations. We propose mobile advertisements displayed within smartphone apps as a way to measure ethnic preferences, by analyzing whether individuals respond disproportionally to particular ethnic cues in the advertisements.

We focus on Bosnia and Herzegovina and the period leading up to the national elections in the fall of 2014, a case where ethnicity should be highly salient. Existing studies suggest that the importance of ethnic identity and ethnic preferences increases before elections [7, 8]. Our approach consists of a measurement experiment where we display mobile advertisements (banners) for an election-related website to smartphone users. These banners have different, randomly selected ethnic cues. We measure variation in the response (click) rate to these cues, where a higher response rate to a particular ethnicity is taken as a measure of preference for that group.

If people respond particularly well to ethnic cues referring to their own group, the share of clicks should be strongly driven by variation in the ethnic demography across the country. For example, in Serb-dominated locations, we should observe a higher share of the clicks for banners with Serb cues. The high spatial resolution of our data allows us to test this relationship at three levels of analysis: (i) the two federal entities, (ii) the municipalities of Bosnia and Herzegovina, and (iii) the neighborhoods of Sarajevo. Against our expectations, though, we find a (weak) trend of preferences towards the ethnic *minority* in a given spatial unit. At the same time, there is tremendous variation in the strength of this effect across different locations in Bosnia and Herzegovina. In the following, our paper introduces the design of our experiment, presents the results and discusses the potential of geo-referenced mobile advertisements for social science applications.

## Methods

### Ethics Statement

The study plan was submitted to the Institutional Review Board (IRB) of the University of Konstanz, Germany, on August 15, 2014. After an initial review, the IRB deemed it unnecessary to conduct a full review and issued a waiver of ethical approval on August 18, 2014. No identifying personal information (e.g. people’s phone numbers or IP addresses) was collected, which is why we were not able to debrief participants. The display of banners within apps and the collection of location data is standard practice in mobile advertising [9]. It is applied to a large number of countries and requires no country-specific permission. By installing the respective app, users agree to see banner displays and share their coordinates (if they refuse, the app either does not work or works only with a reduced set of features). Only users whose settings allowed the app to share their coordinates could participate in our study. Also, the content of our banners (advertisements for a vote matching tool) are ethically unproblematic, since these advertisements were also displayed using other channels in Bosnia and Herzegovina such as news websites. In order to make it impossible to identify participants in our experiment from the geographic coordinates in our data (which would be extremely difficult, but potentially possible in remote areas with very few smartphone users), we nevertheless make the data available only at an aggregated level. We provide sufficient information in S1 Appendix such that others can implement our approach using their own data.

### Research Design

**Fig 1:**
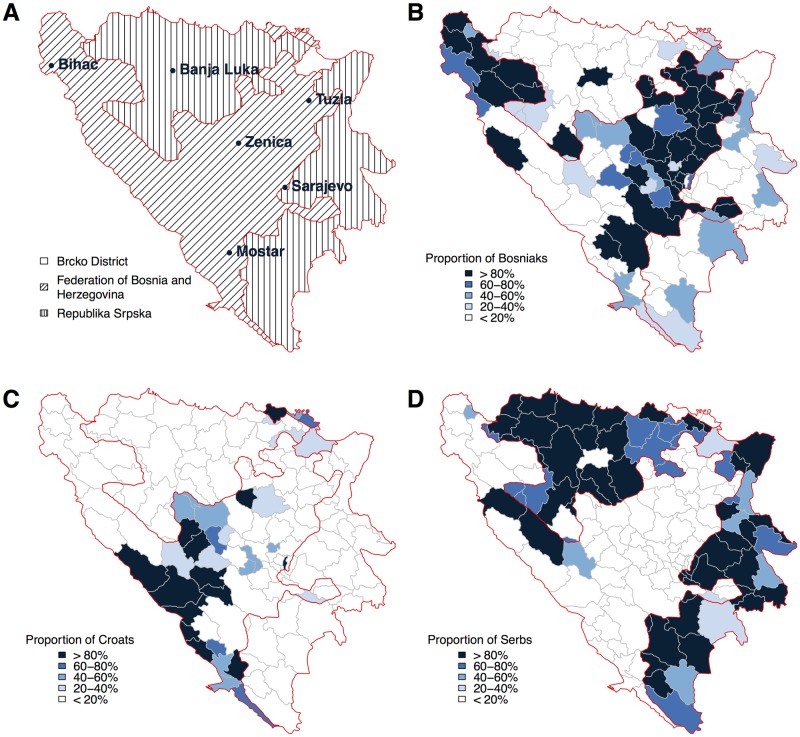
The administrative and ethnic makeup of Bosnia and Herzegovina. (A) The three administrative entities of Bosnia and Herzegovina (Brcko District, Federation of Bosnia and Herzegovina, and Republika Srpska), as well as the largest six cities of the country. The proportion of self-declared Bosniaks (B), Croats (C) and Serbs (D) across 143 municipalities in Bosnia and Herzegovina. Data from the 2013 Bosnian census [12] and the GADM dataset [13].

We implement our experiment in Bosnia and Herzegovina, a country where ethnicity plays a major role in politics and the daily life of citizens. The ethnic demography of the country offers excellent opportunities for examining ethnic preferences: the country consists of two main political entities, the *Federation of Bosnia and Herzegovina* where ethnic Bosniaks constitute the majority, and the *Republika Srpska* with a Serb majority (as well as the very small *Brcko District*). There are important Croat and other smaller minorities throughout the country. Fig 1 displays the three administrative entities (panel A) and the distributions of the three main ethnic groups: Bosniaks (panel B), Croats (panel C) and Serbs (panel D). A study on the neighboring country Croatia indicates that mobile advertisement is a thriving industry throughout the region [10]. Data from the Bosnian Communications Regulatory Agency [11] suggest that an important share of the Bosnian population (around 30%) can be reached by our mobile advertisements. In fact, this number is much larger than traditional Internet users connecting to the Internet via PCs, since the number of (wired) Internet users in Bosnia and Herzegovina is comparably low.

**Fig 2:**
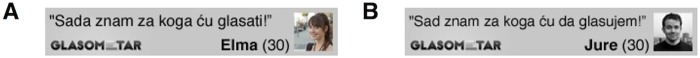
Mobile banners used in the experiment. Two banners used in the experiment, displaying (A) a Bosniak woman, and (B) a Croat man.

We conduct the experiment in the context of the general elections held on October 12, 2014. As mentioned above, this allows us to test our procedure in a most likely case: based on existing research [7, 8], we expect that ethnic preferences should be especially high in the context of elections. We cooperate with a Bosnian NGO and use our banners to promote a vote matching tool designed by this organisation. Vote matching tools, also called voter advice applications, are short online questionnaires about key political issues. The answers of respondents are then matched to the positions of political parties. This approach is used to inform and educate voters about policy positions of political parties, and to eventually help them choose a party to vote for. Examples of vote matching tools in other countries are isidewith.com (US) and votematch.org (UK). Whenever users click on one of our the banners, they are redirected to the website of the vote matching tool. As experimental stimuli, we develop different versions of these banners, which are randomly displayed to app users. Each banner has three elements: (i) the logo of the vote matching tool, (ii) a short sentence (*“Now I know whom to vote for!”*), and (iii) a photo of a person together with a name (see Fig 2). We varied the ethnic cue of the banners through the person’s name and slight spelling differences in the text. The signaling of an individual’s ethnicity by the name is a strategy that has been used by other authors [6, 14]. Obviously, the selection of names is crucial; in order to select names with the highest discriminatory power, we implemented an online survey prior to the experiment (details in S1 Appendix, Section B). The person’s gender was randomly selected with uniform probability. With three ethnic groups and two genders, the experiment included a total of six banner variations.

The mobile advertisement banners were displayed inside smartphone apps. The apps used for this experiment are small utilities for a variety of purposes; for example, they display weather forecasts, stream music or let the user play games. The apps are free of charge to the user, but generate profit through the display of advertisements. When opened, the apps register with an international auction platform where available banner space is sold within milliseconds to the highest-bidding advertiser. This procedure, also called real-time bidding (RTB), resembles automated stock exchanges and is the most common way of selling ad space in the online advertising industry [15]. For the purpose of this experiment, we cooperated with a company specializing in geo-referenced mobile advertising. The company was responsible for managing the entire banner delivery service, and randomly displayed one of our six banners whenever banner space became available. This procedure guarantees a temporally and spatially random delivery of the different banner versions. The company also ensured that only users in Bosnia and Herzegovina would be presented with the banners of our study, and collected a number of additional data on each displayed banner (see below). Fig 1 in S1 Appendix plots the number of displays and clicks on a map of Bosnia and Herzegovina.

## Results

Our banners (*N* = 1,150,552) were delivered in two phases: during a first round from September 23 to 30 (*N* = 545,062), and a second round from October 7 to 13, 2014 (*N* = 605,490). Section A of S1 Appendix contains further details on the sample, including the number of apps and unique users. Users were randomly presented with one of six different versions of the banners. For each displayed banner, we obtained data on the precise date and time, the spatial coordinates, the name of the app, the operating system, a unique device id of the user’s smartphone (if available), the version of the banner, and whether it was clicked or not. A first analysis revealed that some of the traffic must have been artificially generated by so-called “bots”, since certain combinations of apps and geographic coordinates showed up with extremely high frequencies. These bots are computer programs emulating online human behavior to generate profit, and are a well-known issue in online marketing [16, 17]. Using a simple heuristic based on frequency and combinations of geographic coordinates and apps, we exclude this data (*N* = 340,118) from our sample (for more detail on the heuristic, see Section C of S1 Appendix). This procedure left us with 810,434 observations, which we assume not to be primarily generated by bots and which the subsequent main analysis is based on.

**Fig 3:**
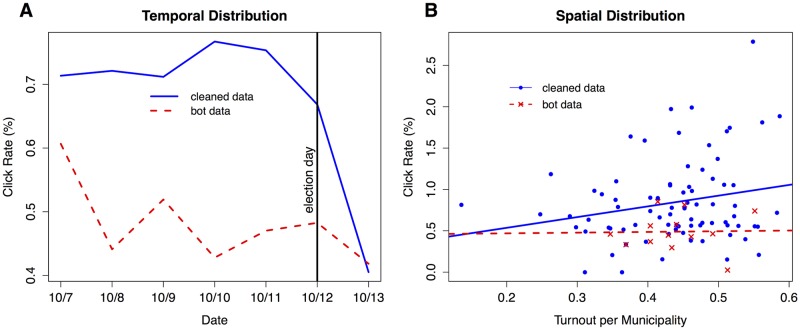
Temporal and spatial distribution of the click rate. Variations in political interest as revealed by (A) the temporal and (B) spatial distribution of the click rate.

We conduct four plausibility checks to test whether our procedure effectively excludes observations generated by bots, while keeping observations generated by humans. Two of these checks are presented here, the other two are found in Section C of S1 Appendix. The plausibility checks are based on the assumption that the frequency with which humans click on political advertisements is influenced by their political interest, while the frequency of clicks generated by bots is not. In other words, we rely on a factor specific to humans (temporal and spatial variations in political interest), which today’s bots are unable to emulate. Since human political interest should be strongly determined by key political events, we expected a higher click rate across Bosnia and Herzegovina as the election day gets closer. For our first test, panel A in Fig 3 displays the daily click rate for cleaned and bot-generated data for the days leading up to the elections. The click rate for the cleaned data increases steadily as the election day approaches, and drops sharply the day after. The click rate for the bot-generated data, on the other hand, does not show any clear trend connected to the elections.

For the second plausibility test, panel B in Fig 3 shows the overall click rate at the municipality level as a function of voter turnout. This test includes only municipalities with at least 500 displayed banners (*N* = 71) and only data from the second round of the experiment, since we assume the correlation between voter turnout and political interest to be more pronounced close to the elections. Interest in political advertisements as measured by the click rate should be higher in municipalities with a higher proportion of politically interested citizens, who will also be more likely to vote. As the figure shows, there is clear evidence in favor of this expectation. The coefficient (*β* = 0.012) for the cleaned data is significant at the 10-percent level in a linear regression. For the bot data, the relationship between turnout and click rate is not significant. Taken together, both plausibility checks presented here suggest that we are able to exclude bot-generated data from our sample with a sufficiently high level of accuracy.

To measure our main quantity of interest, ethnic preference for a particular group, we compute the *majority ethnic click share*. This measure corresponds to the number of clicks on banners displaying a person from the ethnic majority in relation to the total number of clicks in a given spatial unit. For example, Serbs constitute the ethnic majority in East New Sarajevo, and thus the majority ethnic click share for this neighborhood corresponds to the number of clicks on banners displaying a Serb divided by the number of all clicks made by users in this neighborhood. Our approach of measuring ethnic preferences by the click share allows us to exclude heterogeneous overall interest in our banners between spatial units (different click rates). Since our banners differed only in the ethnic cues they contain, differences in the click share were exclusively due to different responses to those ethnic cues, and not to different click rates across different spatial units.

**Fig 4:**
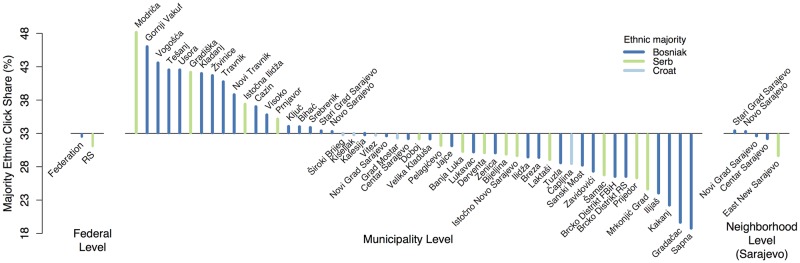
Majority ethnic click share. Deviation of the majority ethnic click share from the expected share of 33%, at three different levels of analysis (federal, municipality, and neighborhood level).

Since our data contain precise geographic coordinates of users, we are able to test our measurement of ethnic preferences at three different levels, comparing (i) the two federal entities, (ii) different municipalities, and (iii) different neighborhoods in Sarajevo. At each level, we test the expectation that banners of the majority ethnic group received the highest ethnic click share, i.e. the highest proportion of clicks. For each spatial unit we examine, the null hypothesis is that the local ethnic configuration has no effect on the ethnic click share, and each ethnicity is clicked at the same rate as the others. This should lead to a majority ethnic click share of 33%. Fig 4 shows the deviation of the respective unit’s majority ethnic click share from this value, for the three levels of analysis. If, according to our expectation, users are primarily responding to banners of the local ethnic majority, the ethnic click share for the majority should be consistently *above* the value of 33%.

### Federal Level

In Fig 4 (federal level), we compare the two main political entities of Bosnia and Herzegovina, the Federation of Bosnia and Herzegovina (Bosniak majority) and the Republika Srpska (Serb majority). We exclude the Brcko District, which is (i) very small, and (ii) ethnically quite mixed. However, the two municipalities of the Brcko District are included in our analysis at the municipality level. As we can see in Fig 1, the majority ethnic click share is below 33% in both cases (32.63 and 31.81%, respectively). Thus, there is a slight tendency in the opposite direction to what we expect: users seem to preferably click on banners displaying a person from an ethnic *minority* in their region. It is interesting to note that the majority ethnic click share *decreases* with the size of the ethnic majority: it is lower in the Republika Srpska (RS), where the Serbian majority makes up approximately 81% of the population as compared to the Federation, where the Bosniak majority corresponds to approximately 71% of the population (numbers on the ethnic set-up of the two entities based on the 2013 Bosnian census [12]).

### Municipality Level

**Fig 5:**
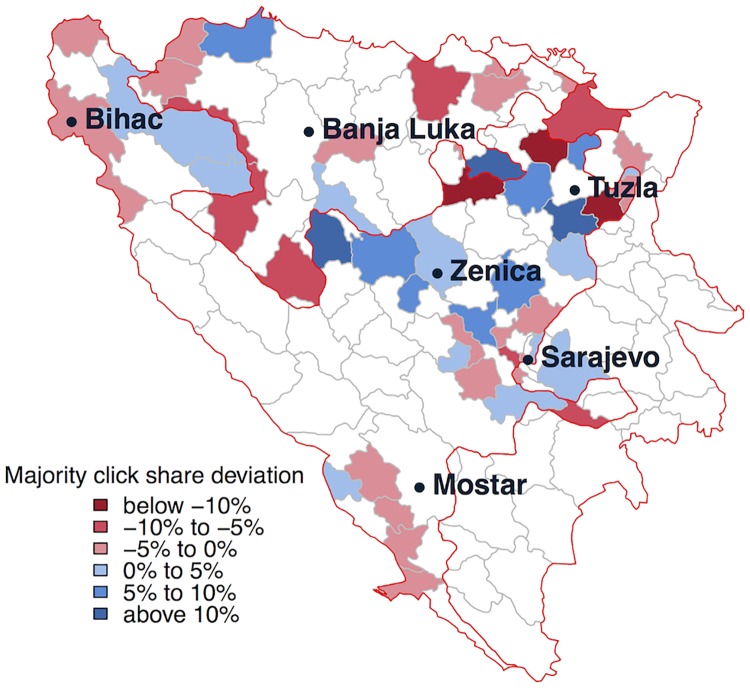
Map of the spatial distribution of the majority ethnic click share. Deviation of the majority ethnic click share from the expected share of 33%, distribution across the municipalities of Bosnia and Herzegovina. Only municipalities with at least 30 clicks are included (*N* = 52).

We use recently published data from the 2013 Bosnian census [12] on the ethnic make-up and data by Hijmans, Garcia and Wieczorek [13] on the geographic boundaries of municipalities in Bosnia and Herzegovina. The 2013 census includes data on the number of individuals belonging to each group, based on the individual’s self-reported ethnicity. We only include municipalities with at least 30 clicks (*N* = 52) to ensure that the click share is a sufficiently reliable measure. The middle panel in Fig 4 displays the majority ethnic click share by municipality. Again, in the larger part (56%) of municipalities, the majority ethnic click share is below 33%, which runs counter to our hypothesis. However, we find no significant effect of the *majority size* on the majority ethnic click share (see Section D of S1 Appendix). Fig 5 displays the information from panel B in Fig 4 on a map. The map suggests no clear clustering of municipalities with a majority ethnic click share below (red) and above 33% (blue). However, it is difficult to draw clear conclusions in this regard, as only 52 municipalities out of 143 had sufficient clicks (at least 30) to be included in the analysis.

### Neighborhood Level

Lastly, we exploit the detailed location data that come with our banners and compare districts within Sarajevo. Bosniaks constitute the ethnic majority in four districts of Sarajevo, but the city has one suburb (East New Sarajevo) populated mainly by ethnic Serbs. Data from the 2013 Bosnian census [12] reveals that Bosniaks constitute between 75% and 88% of the population in the four districts of Sarajevo, while Serbs constitute 96% of the population in the suburb of East New Sarajevo. The right panel in Fig 4 shows the distribution of the majority ethnic click share across the four districts and the suburb of East New Sarajevo. Even on this level of analysis, the majority ethnic click share is below 33% for the larger part of our sample, which indicates that Serbian banners were considered slightly more attractive in the Bosniak parts of Sarajevo, while Bosniak banners were more clicked on in the Serbian suburb.

In sum, there is no clear trend that regions dominated by a particular ethnicity generate more clicks on the majority group. Instead, we observe a weak tendency towards *minority ethnic preferences*: users seem to have a slight tendency of preferring banners from an ethnic minority in a given spatial unit. There could be two main potential explanations for this finding: (i) minority “hyperinterest”, and (ii) surprise effects about minority behavior. Minority hyperinterest would signify that most clicks are caused by a minority particularly interested in ads displaying a member of their group in locations where the minority is underrepresented in public places. However, we believe that our evidence contradicts this (see Section D in S1 Appendix). We therefore consider the second explanation more likely, namely surprise effects about minority behavior. This second explanation is connected with the specific message of our banners (*“Now I know whom to vote for!”*). In Bosnia and Herzegovina, making voting decisions is generally harder for ethnic minorities than for the ethnic majority in a given constituency. Since candidate lists follow ethnic lines, voters can in principle only vote for parties from their own ethnic group. By default, parties representing an ethnic minority have guaranteed seats, but will never get a majority of votes in a given constituency. Therefore, displaying members of an ethnic minority stating that they are now certain about their voting decision may be surprising and generate more interest (both among minority and majority members) than having a member of the ethnic majority sending the same message.

## Discussion

Our proof-of-concept study illustrates how mobile advertising can be used in a measurement experiment to gauge the level of ethnic preferences at a very high temporal and spatial resolution. When adjusting the experimental stimuli, it will be possible to measure a variety of societal variables with a comparable design, as for example variance in issue salience [18, 19]. While there have been other approaches of measuring issue salience relying on modern communication technology such as page view statistics [20] or search engine queries [21, 22], mobile banners allow for a much higher level of geographic resolution.

One of the important advantages of our method is the high flexibility with respect to deployment in terms of time, cost, and the choice of location and experimental stimuli, while at the same time being highly precise as regards randomization of treatment and the location of targeted individuals (for more detail on those factors, see Section E in S1 Appendix). Comparing this approach to other ICT-based experiments, a further advantage is the broad recruitment of subjects; while such experiments will always be restricted to a technologically-connected subset of the population, we can avoid further restricting the pool of subjects to users of certain services such as Amazon’s *Mechanical Turk*.

Still, the method is not without limitations. Most importantly, the response remains dichotomous (click/no click), which requires adequately designed stimuli to be used as advertisements. For specific applications, a dichotomous response without data on additional individual-level variables and a resulting lack of certainty about the underlying mechanisms may pose a problem. Furthermore, fraudulent traffic generated by bots will be a recurrent issue, even though companies and mobile advertisement networks are working to reduce it [16]. Again, bots are not specific to our method, but are an issue for many other ICT-based methods, including page view statistics or Amazon’s *Mechanical Turk* [23, 24]. In contrary to other, non-experimental ICT-based methods, however, our approach allows to somewhat limit the influence of bots on our results: first, the fine-grained location data that comes with our method gives us a way to exclude an important share of bot-generated data. As we show with the plausibility checks presented here, our procedure of excluding bot-generated data worked effectively. Second, bots do not take into account the experimental variation (i.e. ethnic cues) in the banners, and hence generate noise, but not bias. If anything, bots thus make it more difficult to identify an effect. In summary, we therefore believe that for many applications, the advantages of this method well outweigh the shortcomings, especially when it comes to variables such as ethnic preferences or issue salience that are typically hard to measure. We encourage social scientists to further explore measurement experiments based on mobile advertising for their research. For example, rather than setting up a campaign solely for an experiment, researchers could consider piggy-backing on existing advertisement campaigns, which could further reduce costs and make it possible to run such studies over an extended period of time.

## Supporting Information

S1 Appendix Online Appendix for “Measuring Ethnic Preferences in Bosnia with Mobile Advertising”. Contains additional information on research design and analyses.
(PDF)
