# Supplementary material for: Measuring Ethnic Preferences in Bosnia and Herzegovina with Mobile Advertising
Source: PLoS One. 2016 Dec 22;11(12):e0167779. doi: 10.1371/journal.pone.0167779 (PMC5179080; doi:10.1371/journal.pone.0167779)
Supplement: S1 Appendix — Contains additional information on research design and analyses. (PDF) [file pone.0167779.s001.pdf]

Online Appendix for "Measuring Ethnic Preferences in Bosnia with Mobile Advertising"

November 29, 2016

Annerose Nisser[1,2*], Nils B. Weidmann[1]

1 Department of Politics and Public Administration, University of Konstanz, Germany
2 Graduate School of Decision Sciences, University of Konstanz, Germany

* annerose.nisser@uni-konstanz.de

A Description of the Sample

A.1 Banner displays and clicks

In total, our cleaned sample (bot-generated observations excluded based on the heuristics described below) contains 810,434 observations. Banners were displayed in 136 out of the currently 143 Bosnian municipalities. Panel A of figure 1 visualizes the spatial distribution of displays across the country. Municipalities with no banner displays are throughout very small (below 3,500 inhabitants). This signifies that our method is well suited for reaching a large part of the country, but due to its reliance on modern ICT has a bias towards more densely populated areas.

Clicks occurred in 123 out of 143 municipalities, but only 52 municipalities had at least 30 clicks. The spatial distribution of clicks across the country is shown in panel B of figure 1. The municipalities with at least 30 clicks were throughout larger municipalities with on average around 50,000 inhabitants. This means that our method of restricting the sample to municipalities with at least 30 clicks increases the likelihood of people in larger municipalities to be included in the final sample. The overall click rate (clicks as function of displayed banners) was 0.79%, which is a typical value in the field of online marketing.

A.2 Apps and unique device ids

91% of all observations in our cleaned sample contain the name of the app the banner was displayed in. The sample contains 706 different apps, the majority of which are entertainment apps (judging from the name of the apps); we do not offer a detailed analysis of click rate by type of app as we lack theoretical expectations in this regard. However, our data would of course allow for such an analysis.

34% of all observations in our cleaned sample contain a unique device id. In total, our sample contains 14,755 unique device ids. Speaking from this data, each user saw our advertisement on average 18.8 times; and we can estimate that in total about 44,000 unique users were exposed to our experiment. Compared to the number of respondents normally included in surveys, this number is very large.

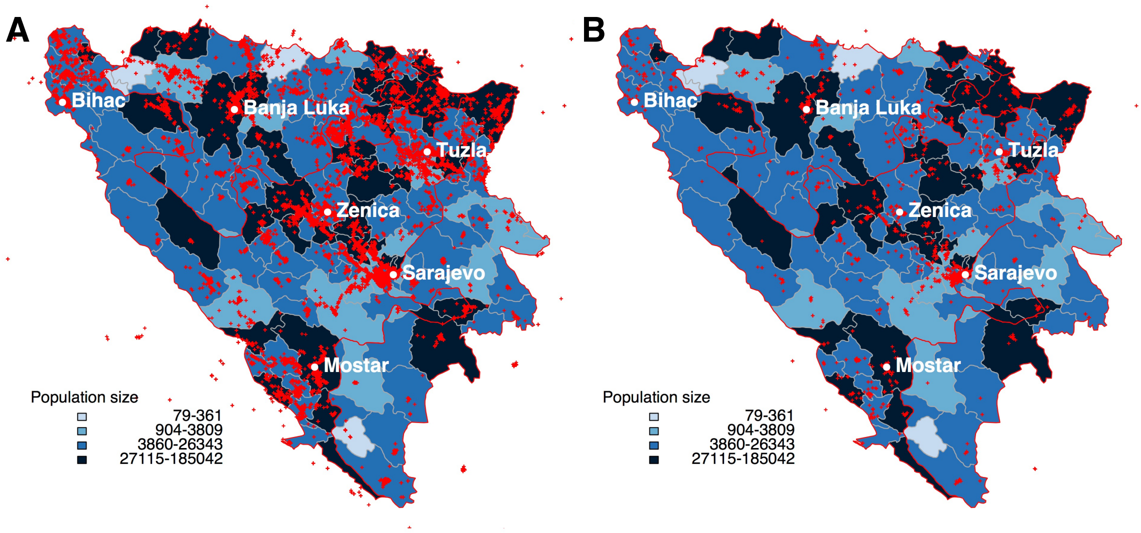

Figure 1: Banner displays (panel A) and clicks (panel B) across the 143 municipalities of Bosnia and Herzegovina. Banner displays and clicks are plotted as red dots and jittered for better display and to protect users' privacy. As the maps indicate, there are by tendency more banner displays and clicks in municipalities with a larger population.

B Treatment: Ethnicity

We use first names and language as ethnic cues on the banners. We selected names with the highest discriminatory power, i.e. names that most clearly signal exclusively one ethnicity. To determine those names, we used a three-step procedure. We first let a native Bosnian create a candidate list of names that correspond to the three ethnicities in Bosnia and Herzegovina. Second, this list was confirmed and further restricted in an informal way with the help of locals. Third, we chose from the resulting list three names for each experimental variation, which resulted in 18 options (3 names x 3 ethnicities x 2 genders = 18 options). Those options were evaluated using an online survey with 42 respondents in Bosnia and Herzegovina. Those respondents were recruited via social media, which allows us to assume that the respondent sample is similar to the smartphone users being exposed to our banners. During the online survey, respondents were asked to guess the ethnicity of persons merely from their names. To eliminate the risk of sequence effects, names and ethnicities to choose from ("Bosniak", "Croatian", "Serbian", "Other", "Cannot tell") were presented in random order to respondents. Tables 1 to 3 show the results from the survey. We chose the following names: *Elma* and *Emir* (Bosniak female and male), *Matea* and *Jure* (Croatian female and male), and *Milica* and *Nemanja* (Serbian female and male).

	Elma	Selma	Ajla	*Emir*	Haris	Advan
Bosniak	*35*	36	33	*36*	33	28
Serbian	0	0	0	0	0	0
Croatian	0	0	0	0	0	0
Cannot tell	0	0	0	0	1	5
dropout	4	4	5	4	4	4
no answer	2	2	2	2	3	3
Other	1	0	2	0	1	2
Sum	42	42	42	42	42	42

Table 1: Results for Bosniak female and male names from the survey.

	Matea	Martina	Anita	*Jure*	Marin	Mateo
Croatian	*31*	30	21	*35*	36	31
Bosniak	0	0	9	0	0	0
Serbian	1	0	0	0	0	1
Cannot tell	2	4	2	0	0	1
Other	1	2	3	0	0	2
no answer	2	2	2	2	2	2
dropout	5	4	5	5	4	5
Sum	42	42	42	42	42	42

Table 2: Results for Croatian female and male names from the survey.

	Milica	Jelena	Jovana	*Nemanja*	Uroš	Stefan
Serbian	*34*	25	35	*33*	26	31
Croatian	0	2	0	1	2	3
Bosniak	0	0	0	2	0	0
Other	0	0	1	0	4	0
Cannot tell	2	6	0	0	3	2
no answer	2	4	2	2	3	2
dropout	4	5	4	4	4	4
Sum	42	42	42	42	42	42

Table 3: Results for Serbian female and male names from the survey.

C Bots and Fraudulent Traffic

Our detection routine for fraudulent web traffic is based on the frequency and combinations of geographic coordinates and apps. As seen in table 4 and 5, certain combinations of coordinates and apps appeared with extremely high frequency. For example, table 4 shows that 16% (89,096) of all observations of the first round ($N = 545,062$) shared the same latitude (18.3833). In the second round ($N = 605,490$), the five most frequent combinations of geographic coordinates were produced by only one app each (see table 5). Such patterns do not appear to be generated by real users.

As a result, we use the heuristic of excluding all observations of banners displayed in apps containing the names "music entertainment", "Music Entertainment" or "Premium" in both the first and second round, and excluding the five most frequent geographic coordinates in the second round. We define that sample as *cleaned data* ($N = 810,434$), as opposed to *bot-generated data* ($N = 340,118$)). To test our heuristic for excluding non-human traffic, we run four plausibility checks: we examine (1) the distribution of banner displays by population, (2) by time of the day and (3) the temporal and (4) spatial distribution of political interest. Plausibility checks (3) and (4) are presented in the main article, results for checks (1) and (2) are presented in the following paragraphs.

Rank	Coordinates	Frequency coordinate	Apps using these coordinates	Frequency combination
1	18.3833_43.85	54664	*"Premium Symbian music entertainment 320x50"*	29382
			"Premium Windows Phone Music Entertainment 320x50"	20782
			... and others ...	
2	18.3833_43.850006	34431	*"Water Puzzle - Android App - 320x50"*	11440
			"Android App -Social - DL - Music 320x50 (Waterfall)"	3397
			... and others ...	
3	18_44	28238	*"Premium Symbian music entertainment 320x50"*	13057
			"Premium Windows Phone Music Entertainment 320x50"	6631
			... and others ...	
4	17.80233_44.14415	26615	*"Dino Assassin Racing - Android - 320x50 - Top Tier"*	26615
5	17.8081_43.3433	18745	*"Premium Symbian music entertainment 320x50"*	12611
			"Premium Windows Phone Music Entertainment 320x50"	5477
			... and others ...	

Table 4: The most frequent combinations of longitudes and latitudes from the first round (total number of observations in the first round $N = 545,062$).

Rank	Coordinates	Apps using these coordinates	Frequency combination
1	18.42683_43.89189	*"Animal Faces Photography - Android - 320x50 - Top Tier"*	38002
2	18.65621_44.54054	*"Geometry Crash Family Entertainment"*	36872
3	18.14225_44.43243	*"Selfye Camera Photography - Android - 320x50 - Top Tier"*	29921
4	18.695147_44.53003	*"Fat Piggy Run air.com.academmedia.FatPiggyRun"*	26207
5	18.01273_44.66667	*"Make Me Girl Photography - Android - 320x50 - Top Tier"*	19609

Table 5: The most frequent combinations of longitudes and latitudes from the second round (total number of observations in the second round $N = 605,490$).

C.1 Banner displays by population

Figure 2 shows the number of banner displays per municipality as a function of population size and data type (separately for cleaned and bot-generated data). Data on the population size in a given municipality was obtained from 2013 Bosnian census [1]. The figure indicates that more banners were displayed in more populated municipalities, both when using cleaned and bot-generated data. However, the variation in banner displays is much better explained by population size for the cleaned data ($R^2 = 0.573$) compared to bot-generated data ($R^2 = 0.155$). Furthermore, bot-generated data was displayed only in 71 municipalities, while cleaned data was displayed in 136 out of a total of 143 municipalities. This indicates that bots tend to be more geographically "clustered" than real, human users.

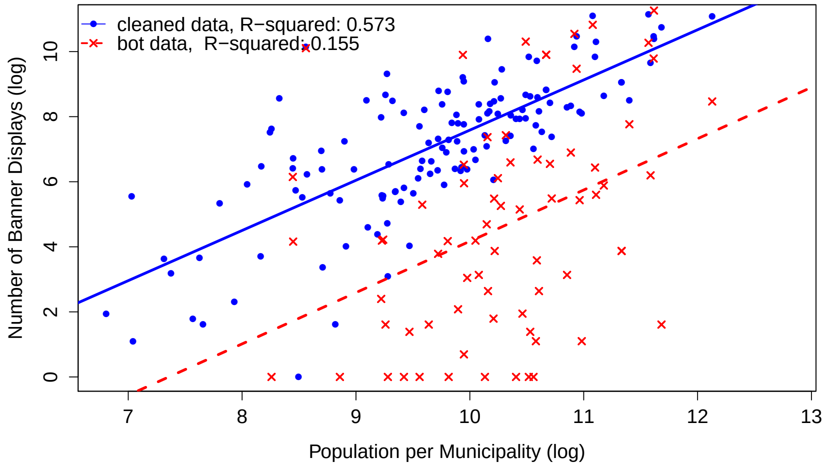

Figure 2: Number of banner displays per municipality as function of population size and type of data (cleaned vs. bot), population data obtained from the 2013 Bosnian census [1], $N = 136$ for cleaned data, $N = 71$ for bot-generated data.

C.2 Banner displays by time of day

As another indicator of "human activity", we expect that less banners are displayed during nighttime, when less people are online. At the same time, we expect the variation between day and night to be less pronounced for bots, which should not have a typically human sleep-wake cycle. Bots should be actively opening apps even during nighttime, when a majority of human users is sleeping. This expectation is supported by our data. In the cleaned data, 82% of all banners were displayed not earlier than 5am and before 11pm. If all banner displays were equally distributed between day and night, only 75% of all banners should have been displayed during that time (18 hours/24 hours=75%). Figure 3 shows the distribution of banner displays as a function of daytime. For bot-generated data, the percentage of displayed banners seems to be more equally distributed across 24 hours, while the cleaned data (which we assume to go primarily back to "human traffic") fluctuates more. This is in line with findings from the Association of National Advertisers [2], which suggest that only 50% of bots are sophisticated enough to keep daylight hours. Furthermore, our finding is in line with evidence from the Association of National Advertisers [2] that bot percentages are higher during night hours.

Comparing this variation with the variation seen in the click rate leading up to the elections and across municipalities (figure 3 in the main article), it seems that some bots are probably programmed to emulate human behavior with regard to waking patterns; but they are clearly not sophisticated enough to react to external events based on the content of advertisements.

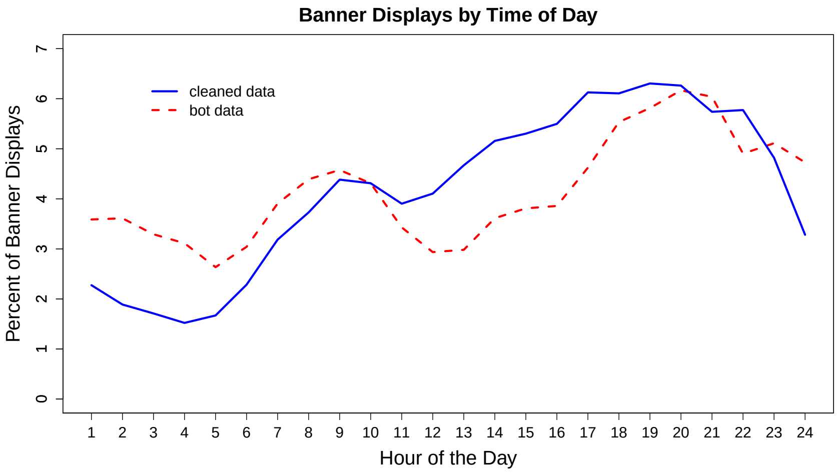

Figure 3: Displayed banners as a function of daytime in the first and second round for bot and cleaned data.

D Who Clicks? – Problem of Ecological Inference

One major difficulty of our research design regards the fact that we have no information on the socio-demographic characteristics of those who view and eventually click the advertisements. Potentially, there are unobserved characteristics such as gender or household income explaining why people do or do not click on one our advertisements. – However, it is important to remember in this context that we are not interested in *absolute*, but only in *relative* click shares. In other words, we are interested in *variations* in the click share, and individual background characteristics of smartphone users cannot explain those variations (unless there are very uneven spatial distributions of the background characteristics of smartphone users; which we do not expect to be the case). Our measurement experiment allows us thus to discern whether ethnic preferences can explain variations in the click share among the population of smartphone users in Bosnia and Herzegovina.

Another potential concern regards whether clicks may first and foremost be caused by a "hyper-interested" minority. In this scenario, the ethnic majority clicks little to nothing on the banners. On the other hand, the ethnic minority – potentially undecided on whom to vote for – feels much more appealed by the banners, and specifically by banners displaying members of their own (minority) ethnic group. When regressing the relative size of the ethnic minority (between 0 and 0.5) on the click rate, we find no significant effect (table 6). Furthermore, we find no significant effect of the size of the ethnic minority on the minority ethnic click rate (= clicks on banners displaying the ethnic minority divided by the number of all clicks, table 7). This signifies that the size of the ethnic minority in a municipality does not influence how many clicks "minority banners" receive. If we assume that "hyperinterest" among the minority does not increase disproportionally when the size of the minority is smaller, this can be interpreted as evidence against the hypothesis that clicks are mainly generated by a hyperinterested minority.

Because minority and majority shares of the population and clicks each sum up to 1, this signifies also that the majority size has no significant influence on the majority ethnic click share.

	Estimate	Std. Error	t value	Pr(>|t|)
(Intercept)	0.0107	0.0014	7.55	0.0000
Minority size	0.0022	0.0054	0.40	0.6896
Croat majority	-0.0015	0.0030	-0.50	0.6177
Serb majority	-0.0001	0.0019	-0.03	0.9772

Table 6: Regression of the click rate on the minority size (in percent), only municipalities with at least 30 clicks, $N = 52$.

	Estimate	Std. Error	t value	Pr(>|t|)
(Intercept)	0.6704	0.0155	43.16	0.0000
Minority size	-0.0081	0.0596	-0.14	0.8921
Croat majority	0.0099	0.0327	0.30	0.7643
Serb majority	0.0041	0.0209	0.19	0.8464

Table 7: Regression of the minority ethnic click share on the minority size (in percent), only municipalities with at least 30 clicks, $N = 52$.

E Costs and Time Frame

As we mention in the article, our method has advantages with regard to costs and time necessary for preparation. After having found suitable cooperation partners, namely a company specialized in geo-located smartphone advertisement and a Bosnian NGO, the experiment took about three weeks of intense preparations. For displaying the banners, we invested a limited budget of about $5,000. Comparing this to a survey implemented in 120 municipalities in Bosnia and Herzegovina with more than 40,000 participants, the cost and time advantages are obvious. At the same time, our results remain of course ambiguous, which makes it necessary to further develop the approach for future applications.

References

[1] Agency of Statistics of Bosnia and Herzegovina. Results of the 2013 Census of Population, Households and Dwellings in Bosnia and Herzegovina; 2016. Available: http://www.popis2013.ba. Accessed 18 September 2016.

[2] Association of National Advertisers. The Bot Baseline: Fraud in Digital Advertising; 2014. Available: http://www.ana.net/content/show/id/botfraud. Accessed 8 December 2015.
